# Effects of media stories featuring coping with suicidal crises on psychiatric patients: Randomized controlled trial

**DOI:** 10.1192/j.eurpsy.2021.2244

**Published:** 2021-11-04

**Authors:** T. Niederkrotenthaler, J. Baumgartner, A. Kautzky, M. Fellinger, R. Jahn, A. Wippel, M. Koch, D. König-Castillo, A. Höflich, R. Slamanig, A. Topitz, J. Wancata, B. Till

**Affiliations:** 1 Unit Suicide Research & Mental Health Promotion, Department of Social and Preventive Medicine, Center for Public Health, Medical University of Vienna, Vienna, Austria; 2 Wiener Werkstaette for Suicide Research, Vienna, Austria; 3 Clinical Division of Social Psychiatry, Department of Psychiatry and Psychotherapy, Medical University of Vienna, Vienna, Austria

**Keywords:** Lived experience, media, Papageno effect, randomized controlled trial, suicide

## Abstract

**Background:**

Accumulating evidence suggests beneficial effects of media stories featuring individuals mastering their suicidal crises, but effects have not been assessed for psychiatric patients.

**Methods:**

We randomized *n* = 172 adult psychiatric patients (*n* = 172, 97.1% inpatients) to read an educative article featuring a person mastering a suicidal crisis (*n* = 92) or an unrelated article (*n* = 80) in a single-blind randomized controlled trial. Questionnaire data were collected before (*T*_1_) and after exposure (*T*_2_) as well as 1 week later (study end-point, *T*_3_). The primary outcome was suicidal ideation as assessed with the Reasons for Living Inventory; secondary outcomes were help-seeking intentions, mood, hopelessness, and stigmatization. Differences between patients with affective versus other diagnoses were explored based on interaction tests.

**Results:**

We found that patients with affective disorders (*n* = 99) experienced a small-sized reduction of suicidal ideation at 1-week follow up (mean difference to control group [MD] at *T*_3_ = −0.17 [95% CI −0.33, −0.03], *d* = −0.15), whereas patients with nonaffective diagnoses (*n* = 73) experienced a small-sized increase (*T*_2_: MD = 0.24 [95% CI 0.06, 0.42], *d* = 0.19). Intervention group participants further experienced a nonsustained increase of help-seeking intentions (*T*_2_: MD = 0.53 [95% CI 0.11, 0.95], *d* = 0.19) and a nonsustained deterioration of mood (*T*_2_: MD = −0.14 [95% CI −0.27, −0.02], *d* = −0.17).

**Conclusions:**

This study suggests that patients with affective disorders appear to benefit from media materials featuring mastery of suicidal crises. More research is needed to better understand which patient groups are at possible risk of unintended effects.

## Introduction

The question of how to harness protective potentials of media portrayals for suicide prevention has received increasing attention in recent years. The World Health Organization (WHO) emphasizes the role of media as an integral part of suicide prevention strategies [1]: First, there is good evidence that some media portrayals of suicide have harmful effects in that they can trigger further suicides, the so-called Werther effect. In a recent meta-analysis, members of this author group showed that particularly the reporting on suicides by celebrities was associated with a 13% increase in suicides in the 1–2 months after the celebrity death, making media one of the potentially most powerful societal agents in suicide [2]. Second, the relevance of raising awareness of suicide prevention in order to educate the public has been noted in suicide prevention plans globally [1]. In this context, media stories of individuals mastering their suicidal crises can reduce suicidal ideation, the so-called Papageno effect [3]. The first study of the Papageno effect showed that news reports about individuals who contemplated suicide but managed to master their suicidal crises were associated with a decrease in subsequent suicides [3]. A few randomized controlled trials have been conducted to test the Papageno hypothesis, using suicidal ideation as the primary outcome. These studies have all been conducted in the general population or online samples, and suggest a reduction of suicidal ideation during exposure to media items featuring hope and recovery [4–7]. Albeit psychiatric disorders, most importantly affective disorders, are important risk factors for suicidal ideation and behavior, there are currently no data about media effects of stories of mastering suicidal crises from psychiatric patients. About a half to two-thirds of suicides occur among patients with mood disorders [8].

In the present study, we aimed to explore the effect of a media story featuring a recovery from a suicidal crisis in a mainly inpatient psychiatric setting. We hypothesized that patients reading a story of recovery from suicidal ideation would experience a reduction in suicidal ideation compared to a control group. Further, we explored the impact of having an affective versus other diagnosis on the intervention effect, and we assessed gender differences.

## Methods

A single-blinded randomized controlled trial was conducted between February 2, 2018, and August 7, 2020. We recruited patients of the Clinical Division of Social Psychiatry, Department of Psychiatry and Psychotherapy of the Vienna General Hospital and the Department of Internal Medicine of the Hospital Penzing in Vienna, Austria. Exclusion criteria were: health status insufficient (as evaluated by the treating physician), ICD-10 diagnosis F00–F09, inability to communicate or being at imminent risk of self-harm or harming others.

Participants were randomized to read a two-pages newspaper article. In both the intervention and the control group, the gender of the protagonist featured in the article was matched with the gender of the participant to maximize identification. Participants of the intervention group read an interview with an individual with lived experience of suicidal ideation. In this text, the protagonist first talks about his/her suicidal crisis and describes the circumstances that lead to the crisis. The protagonist then describes how he/she became increasingly suicidal, including preparing for a suicide attempt. However, shortly before the act, he/she decides to call the telephone crisis line. The main part of the text then focuses on how he/she worked with the counselor and was ultimately able to overcome the crisis. Participants in the control group received a similar newspaper article in terms of style and length, but unrelated to mental health. In this narrative, the protagonist described his/her citizens’ environmental initiative aiming to rebuild a railway track on the shoreline of a lake. Both articles also included a brief promotion of the telephone suicide prevention crisis line, in order to provide a minimal intervention also to patients in the control group.

Participants provided consent and completed a baseline questionnaire (*T*_1_) on the primary outcome variable (i.e., suicidal ideation) and all secondary outcome variables (i.e., mood, hopelessness, help-seeking intentions, and stigmatizing attitudes toward suicidal behavior). The treating physician conducted a global assessment of functioning. Data on all outcome variables were collected again immediately after reading the article (*T*_2_) and 1 week (±2 days) later (*T*_3_). Additional data were extracted from the medical records, including all psychiatric diagnoses. At *T*_3_, all participants were debriefed. See Supplementary Text 1 for details on power analysis, randomization, and questionnaires used.

We examined the effect of the intervention at *T*_2_ and *T*_3_ with two-way analyses of covariance (ANCOVA), with group assignment (intervention vs. control group) and diagnosis of an affective disorder (F30-F39: yes vs. no) as between-subject factors. All ANCOVAs were controlled for the respective outcome score at baseline (*T*_1_), age, and for the variable “inpatient vs. outpatient.” Individual paired sample *t*-tests were used for comparisons with baseline (mean change from baseline) and Bonferroni-corrected contrast test for comparisons between study groups (mean difference between intervention and control group). Gender differences were explored with separate models that included respective interaction terms (i.e., group assignment × gender) [9]. The Ethics Board of the Medical University of Vienna approved the study (see Supplementary Text 2).

## Results

Supplementary Figure S1 illustrates the study flowchart. A total of *n* = 172 individuals were randomly allocated either to the intervention (*n* = 92) or the control group (*n* = 80). *N* = 153 participants (89.0%) completed the entire survey. Mean age was 38.1 years (*SD*: 15.1), *n* = 99 were women (57.6%), *n* = 73 were men (42.4%). *n* = 99 patients (57.6%) were diagnosed with an affective disorder (F30–39). An overview of participant characteristics is provided in Supplementary Table S1. The main results are provided in Table 1.

**Table 1.** Suicidal ideation after article exposure (*T*_2_) and 1 week later (*T*_3_) as well as secondary outcome variables among all participants and stratified for affective disorder diagnosis.

	Control group (*n* = 80)		Intervention group (*n* = 92)			
	Mean score (95% CI)	Mean change (95% CI)^a^ from baseline	Mean score (95% CI)	Mean change (95% CI)^a^ from baseline	Mean difference (95% CI)^b^	Cohen’s *d* ^b^
Suicidal ideation						
After exposure (*T*_2_)						
Affective disorder	3.30 (3.23, 3.37)	−0.01 (−0.08, 0.06)	3.25 (3.19, 3.32)	−0.07 (−0.13, −0.01)*	−0.05 (−0.15, 0.05)	−0.08 (−0.23, 0.07)
No affective disorder	3.32 (3.24, 3.39)	0.02 (−0.05, 0.09)	3.43 (3.35, 3.51)	0.12 (0.02, 0.21)*	0.11 (−0.001, 0.23)	0.15 (−0.001, 0.30)
Both groups combined	3.31 (3.26, 3.36)	0.01 (−0.04, 0.05)	3.34 (3.29, 3.39)	0.00 (−0.05, 0.06)	0.03 (−0.05, 0.11)	0.06 (−0.09, 0.21)
One week later (*T*_3_)						
Affective disorder	3.37 (3.25, 3.49)	0.07 (−0.05, 0.19)	3.21 (3.10, 3.32)	−0.10 (−0.19, −0.01)*	−0.17 (−0.33, −0.03)*	−0.15 (−0.30, −0.003)
No affective disorder	3.17 (3.04, 3.30)	−0.12 (−0.26, 0.03)	3.41 (3.28, 3.54)	0.12 (−0.04, 0.27)	0.24 (0.06, 0.42)*	0.19 (0.04, 0.34)
Both groups combined	3.27 (3.19, 3.36)	−0.02 (−0.11, 0.08)	3.31 (3.22, 3.39)	−0.01 (−0.10, 0.07)	0.04 (−0.09, 0.16)	0.05 (−0.11, 0.20)
Suicidal ideation: coping beliefs						
After exposure (*T*_2_)						
Affective disorder	2.85 (2.74, 2.95)	−0.02 (−0.10, 0.05)	2.79 (2.70, 2.88)	−0.10 (−0.19, −0.01)*	−0.06 (−0.20, 0.08)	−0.06 (−0.21, 0.09)
No affective disorder	2.82 (2.71, 2.93)	−0.03 (−0.14, 0.07)	2.98 (2.86, 3.10)	0.11 (−0.05, 0.26)	0.16 (−0.003, 0.32)	0.15 (−0.003, 0.30)
Both groups combined	2.83 (2.76, 2.91)	−0.03 (−0.09, 0.03)	2.88 (2.81, 2.96)	−0.02 (−0.10, 0.06)	0.05 (−0.06, 0.16)	0.07 (−0.08, 0.22)
One week later (*T*_3_)						
Affective disorder	2.89 (2.73, 3.06)	0.03 (−0.11, 0.18)	2.66 (2.51, 2.80)	−0.22 (−0.36, −0.08)*	−0.24 (−0.46, −0.02)*	−0.16 (−0.31, −0.01)
No affective disorder	2.69 (2.51, 2.86)	−0.14 (−0.34, 0.06)	2.94 (2.76, 3.12)	0.08 (−0.12, 0.28)	0.26 (0.01, 0.50)*	0.16 (0.01, 0.31)
Both groups combined	2.79 (2.67, 2.91)	−0.05 (−0.17, 0.07)	2.80 (2.69, 2.91)	−0.10 (−0.22, 0.02)	0.01 (−0.16, 0.18)	0.00 (−0.14, 0.16)
Mood						
After exposure (*T*_2_)	2.33 (2.24, 2.41)	−0.03 (−0.11, 0.05)	2.18 (2.10, 2.27)	−1.13 (−0.22, −0.03)*	−0.14 (−0.27, −0.02)*	−0.17 (−0.32, −0.02)
One week later (*T*_3_)	2.30 (2.17, 2.43)	−0.06 (−0.21, 0.10)	2.40 (2.27, 2.52)	0.10 (−0.01, 0.21)	0.10 (−0.08, 0.28)	0.08 (−0.07, 0.23)
Hopelessness						
After exposure (*T*_2_)	3.33 (3.25, 3.41)	−0.02 (0.08, 0.03)	3.32 (3.24, 3.39)	−0.06 (−0.14, 0.03)	−0.02 (−0.13, 0.10)	−0.02 (−0.17, 0.13)
One week later (*T*_3_)	3.16 (3.05, 3.28)	−0.15 (−0.24, −0.05)**	3.19 (3.08, 3.30)	−0.16 (−0.28, −0.03)*	0.03 (−0.14, 0.19)	0.02 (−0.13, 0.17)
Help-seeking: crisis line						
After exposure (*T*_2_)	3.42 (3.12, 3.72)	−0.22 (−0.47, 0.04)	3.95 (3.66, 4.23)	0.28 (−0.04, 0.61)	0.53 (0.11, 0.95)*	0.19 (0.04, 0.34)
One week later (*T*_3_)	3.65 (3.32, 3.99)	0.06 (−0.28, 0.40)	3.48 (3.16, 3.80)	−0.04 (−0.41, 0.34)	−0.18 (−0.65, 0.29)	−0.06 (−0.21, 0.09)
Help-seeking: personal contacts						
After exposure (*T*_2_)	3.66 (3.52, 3.80)	−0.01 (−0.15, 0.13)	3.56 (3.43, 3.70)	−0.13 (−0.25, −0.01)*	−0.10 (−0.29, 0.10)	−0.07 (−0.22, 0.08)
One week later (*T*_3_)	3.67 (3.44, 3.89)	−0.02 (−0.25, 0.20)	3.56 (3.34, 3.78)	−0.19 (−0.43, 0.05)	−0.11 (−0.43, 0.21)	−0.05 (−0.20, 0.10)
Help-seeking: professional help						
After exposure (*T*_2_)	4.12 (3.92, 4.31)	−0.19 (−0.38, −0.01)*	4.33 (4.14, 4.52)	0.01 (−0.18, 0.21)	0.21 (−0.06, 0.49)	0.12 (−0.03, 0.27)
One week later (*T*_3_)	4.37 (4.14, 4.61)	0.08 (−0.12, 0.28)	4.13 (3.91, 4.36)	−0.10 (−0.37, 0.17)	−0.24 (−0.57, 0.09)	−0.11 (−0.26, 0.04)
Stigmatization						
After exposure (*T*_2_)	1.91 (1.82, 2.00)	0.06 (−0.02, 0.14)	1.79 (1.71, 1.88)	−0.07 (−0.15, 0.02)	−0.12 (−0.24, 0.10)	−0.14 (−0.29, 0.01)
One week later (*T*_3_)	1.81 (1.70, 1.93)	−0.01 (−0.13, 0.11)	1.77 (1.66, 1.88)	−0.09 (−0.22, 0.03)	−0.04 (−0.21, 0.12)	−0.04 (−0.19, 0.11)
Stigma: normalization						
After exposure (*T*_2_)						
Affective disorder	2.52 (2.34, 2.69)	0.07 (−0.16, 0.29)	2.30 (2.11, 2.50)	−0.15 (−0.32, 0.03)	−0.26 (−0.50, −0.02)*	−0.17 (−0.32, −0.02)
No affective disorder	2.25 (2.09, 2.41)	−0.13 (−0.28, 0.02)	2.40 (2.21, 2.59)	−0.01 (−0.18, 0.15)	0.10 (−0.18, 0.37)	0.05 (−0.10, 0.20)
Both groups combined	2.41 (2.28, 2.54)	−0.02 (−0.16, 0.11)	2.33 (2.20, 2.45)	−0.09 (−0.22, 0.03)	−0.08 (−0.27, 0.10)	−0.07 (−0.22, 0.08)
One week later (*T*_3_)						
Affective disorder	2.37 (2.15, 2.58)	−0.06 (−0.23, 0.11)	2.28 (2.08, 2.48)	−0.02 (−0.24, 0.19)	−0.09 (−0.38, 0.21)	−0.05 (−0.20, .011)
No affective disorder	2.50 (2.26, 2.73)	0.06 (−0.21, 0.33)	2.38 (2.15, 2.61)	0.02 (−0.30, 0.34)	−0.12 (−0.45, 0.21)	−0.05 (−0.20, 0.10)
Both groups combined	2.43 (2.27, 2.59)	0.00 (−0.16, 0.15)	2.33 (2.18, 2.48)	0.00 (−0.18, 0.18)	−0.10 (−0.33, 0.12)	−0.07 (−0.22, 0.08)

*Note:* *p < 0.05, **p < 0.01 (two-tailed).

a Comparison of means with baseline with paired sample *t*-tests.

b Comparison of means of intervention group with the control group with Bonferroni-corrected contrast tests.

There was a significant group × diagnosis interaction for the primary outcome variable, suicidal ideation at *T*_2_ (*F*[1,157] = 4.85, *p* < 0.05, *η_p_* ^2^ = 0.030) and *T*_3_ (*F*[1,144] = 10.74, *p* < 0.01, *η_p_* ^2^ = 0.069). Participants with affective disorders in the intervention group reported a reduction of suicidal ideation, whereas participants without affective disorders and participants of the control group experienced a slight increase or no change in suicidal ideation. A very similar pattern emerged when the score of the subscale “Survival and Coping Beliefs” was used as outcome variable (Table 1).

There was a significant group main effect for mood at *T*_2_ (*F*[1,160] = 5.08, *p* < 0.05, *η_p_* ^2^ = 0.031). Participants in the intervention group experienced an immediate deterioration of mood, which was not found 1 week after the intervention. Furthermore, there was a significant group main effect for help-seeking intentions from crisis helplines at *T*_2_ (*F*[1,158] = 6.17, *p* < 0.05, *η_p_* ^2^ = 0.038). Participants of the intervention group reported a higher intention of using a crisis helpline in case of a suicidal crisis compared to the control group. Furthermore, there was a group × diagnosis interaction for glorifying and normalizing attitudes toward suicidal behavior close to statistical significance at *T*_2_ (*F*[1,158] = 3.88, *p* = 0.05, *η_p_* ^2^ = 0.024). Participants of the intervention group with affective disorders tended to have lower scores for glorifying/normalizing attitudes immediately after article exposure compared to the control group.

### Gender differences

There were no interaction effects between gender and group assignment for the primary outcome, suicidal ideation, or for any of the secondary outcomes (not shown).

## Discussion

This is the first study that tested media effects of stories featuring mastering of suicidal crises in a psychiatric setting. Patients with affective disorders showed some reduction in suicidal ideation, which was sustained 1 week later. In contrast, patients with other diagnoses showed a small increase in suicidal ideation compared to the control group that was just short of statistical significance. The beneficial effect in depressed patients appears partially consistent with one previous trial conducted among young people from the general population, which indicated that participants with some degree of depressive symptoms showed beneficial effects when exposed to a TV broadcast featuring a young woman speaking about how she recovered from depression. In that study, the narrative of the patient story was focused on depression treatment, and improvements were seen for depressive symptoms [5]. There is a noted scarcity of knowledge about how psychiatric morbidity, including specific diagnoses, impact on effects of media stories related to suicide. The present study suggests that media materials that feature individuals mastering their suicidal crises might resonate better with patients with affective disorders as compared to other patient groups. A subgroup analysis according to diagnostic groups did not reveal any clear patterns regarding which groups among nonaffective diagnoses were associated with increases in suicidality, and sample sizes were small for most groups. Future research with larger sample sizes should investigate how different patient groups react to media stories of mastering suicidality.

Importantly, the intervention group also showed some increase in help-seeking intentions from a telephone crisis line that was promoted in the narrative. Although this effect was short-lived, this is an important finding that indicates that media materials might be suited to promote help-seeking after discharge from hospital. During the time immediately after discharge, it is particularly necessary to establish clear pathways to help-seeking for patients in order to avoid gaps in treatment, and potentially, suicides, which tend to cluster after hospital discharge [1]. Further, individuals with affective disorders have also experienced a reduction in normalizing attitudes toward suicide. This finding is of high clinical relevance. Suicide-related media interventions need to balance the opportunity of promoting suicide prevention with the risk of normalizing suicidal behaviors [10]. There are examples in the literature where educative materials have highlighted suicide as an option to cope with adversity and which have been associated with subsequent increases in suicide [2, 11].

All participants received intervention materials that matched their own gender in order to increase identification with the featured protagonist, and the effect on suicidal ideation was comparable for male and female participants. This finding suggests that both, males and females with mood disorders might benefit from media materials featuring a story of hope and recovery.

Important study limitations were that, due to the Covid-19 pandemic, adaptions had to be made to the trial accordingly (see Supplementary Text 3). Further, although diagnoses were extracted for all patients, it was not possible to determine the primary diagnosis. The sample size was relatively small and findings on differences between subgroups require reassessment with larger samples in the future.

Taken together, the present findings suggest that psychiatric patients with affective diagnoses, a group of high priority for suicide prevention due to the large prevalence of mood disorders among suicide decedents, might benefit from media stories of recovery from suicidal crisis.

## Supplementary Materials

To view supplementary material for this article, please visit http://dx.doi.org/10.1192/j.eurpsy.2021.2244. click here to view supplementary material
