# Supplementary material for: Effects of media stories featuring coping with suicidal crises on psychiatric patients: Randomized controlled trial
Source: Eur Psychiatry. 2021 Nov 4;64(1):e70. doi: 10.1192/j.eurpsy.2021.2244 (PMC8668446; doi:10.1192/j.eurpsy.2021.2244)
Supplement: Supplementary file 1 [file S0924933821022446sup001.docx]

**Supplementary appendix to:**

**Effects of media stories featuring coping with suicidal crises on psychiatric patients: Randomized controlled trial.**

**Table of Contents**

**Supplemental text 1:** Details on methodology: Power analysis; randomization; and outcome measures 3

**Supplemental text 2:** Ethics statement 6

**Supplemental text 3:** Changes to the study protocol 7

**Supplemental Figure 1:** Study flowchart 8

**Supplemental Table 1:** Descriptive characteristics for intervention and control group 9

**References for appendix**  10

**Supplemental text 1.** Details on methodology: Power analysis; randomization; and outcome measures

**Power Analysis**

This trial was ended prematurely due to the outbreak of the COVID-19 pandemic; therefore, adaptions to the study protocol (see supplemental text 3) were necessary. Further, we updated the power analysis below after the pandemic's onset to restrict the analysis to the main research question.

A power analysis with G*Power version 3.1.9.7 [1] revealed that an analysis of covariance (ANCOVA) with a 2×2 factorial design (i.e., two study groups: intervention vs. control group; affective vs. no affective disorder) required a minimum sample size of 151 participants to detect small- to medium-sized effects (*f* = 0.23) on suicidal ideation, consistent with previous studies [e.g., 2,3].

**Randomization**

We generated a randomization list with *Research Randomizer* (<https://www.randomizer.org>) to allocate participants either to the intervention or the control group in a 1:1 ratio. A list of numbers (1-400) was randomized so that each number corresponded to either the intervention or the control group. Patients who provided informed consent were assigned to the next available number on the list.

**Primary outcome measure**

**Suicidal ideation.** The Reasons for Living Inventory (RFL) is a 48-item self-report measure to rate beliefs and expectations for *not* dying by suicide (e.g., “I still have many things left to do”) from 1 (not at all important) to 6 (extremely important) [4]. The scale has six subscales, and the subscale of Survival and Coping Beliefs has been shown to be particularly sensitive to media effects [3,5-7]. Mean scores across all items were calculated with higher scores indicating higher suicidality (score range: 1–6; Cronbach’s α = 0.93). We also calculated a mean score for the subscale Survival and Coping Beliefs (Cronbach’s α = 0.96).

**Secondary outcome measures**

**Hopelessness.** We used the German version [8] of the Beck Hopelessness Scale (BHS) [9] consisting of 20 self-report items (e.g., “My future seems dark to me”) rated on a 6-point scale ranging from 1 (very false) to 6 (very true). Mean scores were calculated with higher scores indicating higher hopelessness (score range: 1–6; Cronbach’s α = 0.94).

**Mood.** We assessed mood with the mood subscale of the Affective State Scale [10]. This is a self-report measure using eight adjectives (e.g., “merry”, “sad”, etc.) rated on a scale from 1 (not at all) to 4 (highly). Mean scores across all items were calculated, with higher scores indicating better mood (score range: 1–4; Cronbach’s α = 0.92).

**Help-seeking intentions.** We used a modified version [5,11] of the General Help Seeking Questionnaire [12]. Respondents rated 11 items assessing the likelihood of seeking help in the case of personal/emotional problems or suicidal thoughts from a variety of specified sources (e.g., “parent”, “phone helpline”, etc.) on a scale from 1 (extremely unlikely) to 7 (extremely likely). We calculated scores for help-seeking intentions from personal contacts, professional help, and crisis helplines, with higher scores indicating greater intentions (score range: 1–7; Cronbach’s α = 0.62 – 0.64).

**Stigmatizing attitudes towards suicidal behavior.** We used the Short Stigma of Suicide Scale [13]. Respondents rated eight adjectives (e.g., “cowardly”, “shallow”, etc.) for suicidal individuals on a scale ranging from 1 (strongly disagree) to 5 (strongly agree). This scale consists of three subscales [13], and we calculated mean scores for the subscales “Stigma” and “Glorification/normalization”. Higher scores indicate higher stigma (score range: 1–5; Cronbach’s α = 0.78 – 0.88).

**Additional measures**

**Global functioning.** Participants’ psychological, social, and occupational functioning was rated by the treating physician with the Global Assessment of Functioning (GAF) scale. This scale consists of one item measuring the extent individuals’ day-to-day life is affected by their mental illness on a scale of 0 to 100 [14].

**Socio-demographics.** We asked participants to report their gender (male, female), age, highest completed school level (below high school, high school, university/college), and nationality (Austrian, other).

**Manipulation check**. We asked participants to indicate whether the protagonist in the featured newspaper article has experienced a suicidal crisis (yes, no, don’t know).

**Blinding success.** Consistent with similar previous studies [5-7], we asked respondents to indicate what group they thought they had been allocated to (intervention group, control group, don’t know).

**Supplemental text 2: Ethics statement**

Ethical approval of this study was obtained from the Research Ethics Board of the Medical University of Vienna (study protocol 1306/2014, May 26, 2014) and the Ethics Committee of the City of Vienna (study protocol 19-097-0619, June 13, 2019). All participants provided written informed consent. This study was conducted in accordance with the Declaration of Helsinki and registered with the German Clinical Trial Registry (DRKS00014016, February 13, 2018). Changes to the original study protocol are described in this Supplementary Appendix, p.7.

**Supplemental text 3: Changes to the study protocol**

The following changes were made in the study protocol: On August 31, 2018, based on newly available studies, we added help-seeking intentions to the secondary outcomes, and we updated the sample size calculation (i.e., we added the expected dropout rate to the calculation). We also updated the starting date of patient recruitment. On September 5, 2019, we added a second location (i.e., Otto-Wagner Hospital) to the trial (and adapted the inclusion criteria accordingly) and corrected an error in the sample size calculation (i.e., aimed sample size was increased). On March 8, 2021, we updated recruitment information (the trial was interrupted prematurely due to the first lockdown during the COVID-19 pandemic) as well as statistical protocol and sample size calculation (i.e., limiting data analysis to the primary statistical analysis).

Supplemental Figure 1. Study Flowchart

Randomized (*n* = 172)

Assessed for eligibility (*n* ≈ 215)

## Enrollment

## Analysis

## Follow-Up

## Allocation

Analysed (*n* = 81)

Analysed (*n*= 72)

Discontinued participation before completing the entire survey (*n* = 8)

Discontinued participation before completing the entire survey (*n* = 11)

Allocated to intervention (*n* = 92)

♦ Received allocated intervention (*n* = 91)

♦ Did not receive allocated intervention (*n* = 1)

Allocated to control group (*n* = 80)

♦ Received allocated intervention ( *n*= 80)

♦ Did not receive allocated intervention (*n* = 0)

Excluded (*n* ≈ 44)

♦  Not meeting inclusion criteria (*n* ≈ 44)

♦  Other reasons (*n* = 0)

Supplemental Table 1: Descriptive characteristics for intervention and control group

| **Variable** | **Intervention**  **group** | **Control**  **group** | **χ^2^/*T*** |
| --- | --- | --- | --- |
| Gender |  |  |  |
| Females *n* (%) | 51 (55.4) | 48 (60.0) | 0.37^a^ |
| Age *M (SD)* | 41.03 (15.9) | 34.61 (13.5) | 2.83^c,d^ |
| Highest education |  |  |  |
| College/ University *n* (%) | 18 (19.6) | 14 (17.5) | 0.66^b^ |
| High school *n* (%) | 28 (30.4) | 29 (36.3) |  |
| Below high school *n* (%) | 46 (50.0) | 37 (46.3) |  |
| Not Austrian citizen n (%) | 9 (9.8) | 8 (10.0) | 0.001^a^ |
| Diagnostic group |  |  |  |
| Substance disorder (F10-F19) *n* (%) | 23 (25.0) | 21 (26.3) | 0.04^a^ |
| Psychosis (F20-F29) *n* (%) | 7 (7.6) | 8 (10.0) | 0.31^a^ |
| Affective disorder (F30-39) *n* (%) | 56 (60.9) | 43 (53.8) | 0.89^a^ |
| Neurotic disorder (F40-F48) *n* (%) | 40 (43.5) | 39 (48.8) | 0.48^a^ |
| Personality disorder (F60-F69) *n* (%) | 13 (14.1) | 20 (25.0) | 3.26^a^ |
| Outpatients n (%) | 5 (5.4) | 0 (0) | 4.42^a,d^ |
| Duration current treatment (days) M (SD) | 28.40 (17.8) | 28.28 (20.7) | 0.04^c^ |
| Currently has additional outside therapy n (%) | 35 (38.0) | 23 (28.7) | 1.51^a^ |
| Baseline measures (T_1_) |  |  |  |
| GAF Score *M (SD)* | 58.76 (12.8) | 57.78 (12.3) | 0.51^c^ |
| Suicidal ideation *M* (*SD*) | 3.35 (0.8) | 3.28 (0.8) | 0.50^c^ |
| Suicidal ideation: coping beliefs *M* (*SD*) | 2.96 (1.2) | 2.79 (1.2) | 0.92^c^ |
| Mood *M* (*SD*) | 2.26 (0.7) | 2.44 (0.8) | -1.53^c^ |
| Hopelessness *M* (*SD*) | 3.52 (1.0) | 3.18 (1.0) | 2.15^c,d^ |
| Help-seeking intentions: personal contacts *M* (*SD*) | 3.74 (1.4) | 3.60 (1.6) | 0.64^c^ |
| Help-seeking intentions: professional help *M* (*SD*) | 4.12 (1.5) | 4.46 (1.4) | -1.48^c^ |
| Help-seeking intentions: crisis helpline *M* (*SD*) | 3.51 (2.3) | 3.73 (2.1) | -0.66^c^ |
| Stigmatization *M* (*SD*) | 1.91 (0.8) | 1.78 (0.7) | 1.05^c^ |
| Normalization *M* (*SD*) | 2.35 (0.9) | 2.48 (0.8) | -1.00^c^ |
| Completed survey *n* (%) | 81 (88.0) | 72 (90.0) | 0.17^a^ |

*Note*. Frequencies (*n*), percentages (%), means (*M*), and standard deviations (*SD*) provided for each group, as well as χ^2^ values from χ² tests and *t* values from independent t-tests testing group differences.

^a^χ² test result, *df* =1

^b^χ² test result, *df* =2

^c^independent t-test result

^d^ p < 0.05

**References for appendix**

[1] Faul F, Erdfelder E, Lang AG, Buchner A (2007) G*Power 3: A flexible statistical power analysis program for the social, behavioral, and biomedical sciences. *Behavior Research Methods* 39, 175–191.

[2] Till B, Strauss M, Sonneck G, Niederkrotenthaler T (2015) Determining the effects of films with suicidal content: A laboratory experiment. *British Journal of Psychiatry* 207, 72-78.

[3] Till B, Tran US, Voracek M, Niederkrotenthaler T (2017) Beneficial and harmful effects of educative suicide prevention websites: randomised controlled trial exploring Papageno *v*. Werther effects. *British Journal of Psychiatry* 211, 109-115.

[4] Linehan MM, Goodstein JL, Nielsen SL, Chiles, JA (1983) Reasons for staying alive when you are thinking of killing yourself: The Reasons for Living Inventory. *Journal of Consulting and Clinical Psychology* 51, 276–286.

[5] Niederkrotenthaler T, Till B (2020a) Effects of awareness material featuring depressed and suicidal individuals on an audience with depressive symptoms: Randomized controlled trial. *Journal of Behavior Therapy and Experimental Psychiatry* 66, 101515.

[6] Niederkrotenthaler T, Till B (2020b) Effects of suicide awareness materials on individuals with recent suicidal ideation or attempt: Randomized controlled online trial. *British Journal of Psychiatry* 217, 693-700.

[7] Till B, Arendt F, Scherr S, Niederkrotenthaler T (2019) Effect of educative suicide prevention news articles featuring experts with vs without personal experience of suicidal ideation: A randomized controlled trial of the Papageno effect. *Journal of Clinical Psychiatry* 80, 17m11975.

[8] Krampen G (1994) *Skalen zur Erfassung von Hoffnungslosigkeit (H-Skalen). Deutsche Bearbeitung und Weiterentwicklung der H-Skalen von Aaron T. Beck* [Scales for assessment of hopelessness (H-scales). German adaption and enhancement of the h-scales by Aaron T. Beck]. Hogrefe: Göttingen.

[9] Beck AT, Steer RA (1988) *Manual for the Beck Hopelessness Scale*. Psychological Corporation: San Antonio.

[10] Becker P (1988) Skalen für Verlaufsstudien der emotionalen Befindlichkeit [Scales for longitudinal studies of affective state]. *Zeitschrift für experimentelle und angewandte Psychologie* 35, 345-369.

[11] Till B, Tran US, Niederkrotenthaler T (2020) The impact of educative news articles about suicide prevention: A randomized controlled trial. *Health Communication*. [Epub ahead of print].

[12] Wilson CJ, Deane FP, Ciarroch, J, Rickwood D (2005) Measuring help-seeking intentions: Properties of the General Help-Seeking Questionnaire. *Canadian Journal of Counseling* 39, 15-28.

[13] Batterham PJ, Calear AL, Christensen H (2013) The Stigma of Suicide Scale: Psychometric properties and correlates of the stigma of suicide. *Crisis* 34, 13-21.

[14] American Psychiatric Association (1994) *Diagnostic and statistical manual of mental disorders*. APA: Arlington.
